# Effect of desalted *Salicornia europaea L.* ethanol extract (PM-EE) on the subjects complaining memory dysfunction without dementia: a 12 week, randomized, double-blind, placebo-controlled clinical trial

**DOI:** 10.1038/s41598-020-76938-x

**Published:** 2020-11-16

**Authors:** Woo-Jin Lee, Yong-Won Shin, Da-Eun Kim, Mee-Hyang Kweon, Manho Kim

**Affiliations:** 1 Department of Neurology, Seoul National University Hospital, 101 Daehak-ro, Jongno-gu, Seoul, 110-744 South Korea; 2 Center for Hospital Medicine, Department of Neurosurgery, Seoul, Republic of Korea; 3 Research Center, Phyto Corporation, Seoul, 08826 Republic of Korea; 4 Protein Metabolism and Dementia Research Neuroscience Center, Seoul National University College of Medicine, Seoul, South Korea

**Keywords:** Neurology, Dementia

## Abstract

Desalted *Salicornia europaea L.* (SE) inhibits acetylcholine esterase, attenuates oxidative stress and inflammatory cytokines, and activates neurotrophic pathway. We performed 12-week, randomized, double-blind, placebo-controlled study to evaluate the efficacy of PhytoMeal(a desalted SE)-ethanol extract (PM-EE), in improving the cognitive performance in patients with subjective memory impairment. 63 participants complaining memory dysfunction without dementia (Korean Mini-Mental State Examination [K-MMSE] score ≥ 23) were assigned to PM-EE 600 mg/day or placebo. The cognitive domain of the Alzheimer's disease assessment scale-Korean version (ADAS-K) was set as the primary outcome. After 12 weeks, there was no differences in the changes in the primary outcome or the frequency of adverse events between the groups. In the subgroup analysis for the 30 subjects with mild cognitive impairment (MCI, baseline K-MMSE scores ≤ 28), PM-EE significantly improved the color-reading score of the Korean color-word stroop test (8.2 ± 25.0 vs. − 4.7 ± 13.2, *P* = 0.018). Our findings suggest that PM-EE is safe but might not be effective in this setting of this study. However, PM-EE may improve the frontal executive function in the patients with MCI. Further large-sized studies with longer follow-up period is warranted (trial registration number KCT0003418).

## Introduction

Dementia is a very prevalent geriatric disorder with a substantial clinical impact^1^. Due to the increasing life expectancy, the global incidence and the socioeconomic burden of dementia is rapidly expanding^2^. Although acetylcholine esterase (AchE) inhibitors such as donepezil and galantamine and N-methyl-D-aspartate receptor blockers such as memantine have been established to be effective in improving the symptoms of dementia, they have not been shown to change the long-term outcome of the disease^3–6^. Recent studies have demonstrated that pathological, functional, and structural changes in the brain begin to develop as early as twenty years before the clinical manifestation of dementia becomes evident^7^. Thus, the early recognition and management of the disease is increasingly being recognized to be crucial to improve the clinical course of dementia^8,9^. In this regard, the clinical attention of the pre-dementia disease stages such as mild cognitive impairment (MCI), a status with objective deficit in cognitive function but without impairment of daily activities^10,11^, or subjective memory impairment (SMI), the pre-MCI status that presents with a subjective complaint of memory dysfunction with no clinical evidence of deficit in memory or other cognitive functions^18^, are rapidly increasing. Numerous investigations evaluating the candidate medications to treat MCI or SMI have been performed, but no drug has been established as effective^12–17^.

The two major types of dementia, Alzheimer’s disease (AD) and vascular dementia (VD), share common disease pathophysiology consisting of oxidative stress, chronic neuronal inflammation, and dysfunction in the cholinergic system in the brain^19–23^. Therefore, a broad and robust mechanism of action that widely covers multiple pathological processes while maintaining sufficient safety might be required for a new therapeutic candidate.

*Salicornia europaea* L. (SE) is a halophytic plant species that has recently been recognized for its substantial neuroprotective effects^24–26^. Recently, our study group demonstrated an anti-amnesic effect of the desalted and enzyme-digested SE ethanol extract (SE-EE) using a scopolamine-administered amnestic mouse model^27^. A subsequent study also isolated and identified Acanthoside B from SE-EE as a potential candidate substance and reported that Acanthoside B improved cognitive function in the amnestic mouse model with negligible toxicity^28^. We also demonstrated that Acanthoside B regulates cholinergic function by enhancing AchE inhibitory activity, attenuates oxidative stress and inflammatory cytokines, and activates the neurotrophic tropomyosin receptor kinase B/cAMP response element binding/brain-derived neurotrophic factor (TrkB/CREB/BDNF) pathway^28^, which are all processes involved in the key pathomechanism of both AD and VD.

In this study, we hypothesized that the use of PhytoMeal(a desalted SE)-ethanol extract (PM-EE), would be safe and more effective than placebo in improving cognitive performance in patients who are complaining memory impairment but without dementia and performed a randomized placebo controlled clinical trial to evaluate the efficacy and safety of PM-EE.

## Materials and methods

### Study subjects

In this study, we recruited patients who (1) complained subjective feeling of decrement in memory function and visited the outpatient clinic of the Neurology Department of the Seoul National University Hospital (SNUH), (2) were aged between 50 and 85 years, and (3) were without dementia according to the baseline Mini-Mental State Examination-Korean version (K-MMSE) scores (scores of 23 or higher). We excluded patients who (1) had been admitted to hospital within 3 months of the inclusion time with a diagnosis including malignant neoplasm, acute intracerebral haemorrhage, acute cerebral infarction, acute myocardial infarction, unstable angina, or arrhythmia with congestive heart failure; (2) had a current or past history of schizophrenia or major depressive disorder; (3) had a chronic brain disease that affects cognitive function, such as cerebral infarction, dementia, or parkinsonism; (4) had taken medications such as antipsychotics, tricyclic antidepressants, or other neuroprotective agents within 4 weeks of the time of inclusion; (5) were currently taking vitamin E with a dosage higher than 400 international units per day; (6) had undergone estrogen replacement therapy (excluding topical estrogen) within 2 months of the time of inclusion (due to the potential protective effect of estrogen on the cognitive function^29^); (7) had ingested any food products known to be related to cognitive function within 2 weeks of the time of inclusion; (8) were alcohol abusers or dependent on alcohol; (9) had laboratory abnormalities such as a thyroid stimulating hormone level of ≤ 0.1 uIU/ml or ≥ 10 uIU/ml, serum creatinine level ≥ 2 times higher than the upper normal limit, or serum aspartate aminotransferase or alanine aminotransferse levels ≥ 3 times higher than the upper normal limit; (10) had uncontrolled hypertension with resting blood pressure higher than 160/110 mmHg; (11) had uncontrolled diabetes with a fasting blood glucose level of ≥ 180 mg/dl; or (12) had participated in other clinical trials within 3 months of the time of inclusion or were planning to participate in other clinical trials while participating in the current study. This study was registered at Clinical Research Information Service; Korea Centers for Disease Control and Prevention, Ministry of Health and Welfare, Republic of Korea (KCT0003418, date of registration:10/01/2019). This study protocol and supporting documentation were approved by Institutional Review Board (IRB) of SNUH (H-1711-092-901) and the study was performed in compliance with the SNUH IRB regulations and the International Conference on Harmonisation guideline for Good Clinical Practice. Written informed consent to participate was obtained from all enrolled patients.

### Study design and procedures

The current study was designed as a randomized, double-blind, placebo-controlled experiment. Due to the lack of previous clinical study data that evaluated the effect of PM-EE on cognition, we used a study data that evaluated the effect of using *Panax Ginseng* for 12 weeks on ADAS score changes to estimate the size of the study population^30^. Parameters for the estimation was as follows: allocation ratio 1:1, power 0.8, and α error probability 0.05. The estimation returned 26 participants required in each groups, and study population was set as 30 considering a predicted drop-out rate of 10%. At baseline, we obtained participants’ medical histories, performed physical and laboratory examinations, and administered baseline scores for cognitive evaluations. Participants who met the inclusion criteria were randomized into two groups in a 1 to 1 ratio using a simple randomization method by utilizing SAS randomization program. The random allocation sequence was generated by M-H.K and the details of randomization table were unknown to all researchers who enrolled the participants and the clinicians who evaluated the patients. The groups were assigned to receive PM-EE 600 mg/day (two 150 mg tablets, twice daily) or placebo (two placebo tablets, twice daily) for 12 weeks. In the placebo tablets, desalted SE extract substituted with the equal mass of crystalline cellulose and the PM-EE and the placebo tablets were identical in appearance. The details of the contents of raw materials in PM-EE and Placebo tablets are described in Supplemental Table 1. Randomization was performed at the SNUH Clinical Research Unit using a list of computer-generated random numbers. At 6 weeks after treatment, participants were followed up with physical and laboratory examinations and were checked for the development of any adverse events. At 12 weeks after treatment, participants were followed up again with physical and laboratory examinations, were checked for the development of any adverse events, and performed the follow-up cognitive evaluations. The use of medications included in the exclusion criteria was not allowed during the trial.

**Table 1:** Demographic and baseline clinical characteristics.

	PM-EE (n = 26)	Placebo (n = 27)
Female sex (%)	23 (88.46)	22 (81.48)
Age (years)	58.96 ± 6.26	61.70 ± 8.07
Body weight (kilograms)	58.82 ± 6.45	61.72 ± 8.09
Hypertension (%)	4 (15.38)	4 (14.81)
Diabetes (%)	2 (7.69)	3 (11.11)
Hyperlipidaemia (%)	3 (11.54)	5 (18.52)
**Regularly drinks**		
No (%)	22 (88.47)	15 (62.97)
< 1 bottle/week (%)	3 (11.54)	5 (18.52)
1–2 bottles/week (%)	0 (0.00)	3 (11.11)
≥ 3 bottles/week (%)	0 (0.00)	2 (7.41)
**Regularly exercises**		
No (%)	4 (15.38)	8 (29.63)
1–2 times /week (%)	5 (19.23)	2 (7.41)
3 times /week (%)	8 (30.77)	11 (40.74)
4–5 times /week (%)	5 (19.23)	5 (18.52)
Everyday (%)	4 (15.38)	1 (3.70)
**Smoking habits**		
Non-smoker (%)	24 (92.31)	22 (81.48)
Ex-smoker (≥ 6 months %)	2 (7.69)	4 (14.81)
Current Smoker (%)	0 (0.00)	1 (3.70)
**Subjective severity of stress in daily life**		
No stress (%)	3 (11.54)	6 (22.22)
Mild (%)	19 (73.08)	17 (62.96)
Moderate (%)	3 (11.54)	3 (11.11)
Severe (%)	1 (3.85)	1 (3.70)
K-MMSE	27.24 ± 2.20	28.08 ± 1.78

The data are reported as the mean ± standard deviation. K-MMSE: Korean version of the Mini-Mental State Examination.

### Measurements

The K-MMSE was evaluated for baseline cognitive function. At baseline and at 12 weeks after treatment, scores on the Korean version of the Alzheimer's Disease Assessment Scale (ADAS-K) were used to evaluate the global function^31^; scores on the cognitive domain of the ADAS-K (ADAS-cog) the Korean version of the Consortium to Establish a Registry for Alzheimer's Disease (CERAD-K) assessment packet^32^, and the Korean version of the Color-Word Stroop Test (K-CWST) were used to evaluate cognitive function^33,34^; and scores on the Alzheimer Disease Cooperative Study assessment for the Activities of Daily Living (ADCS-ADL) to evaluate the ability to perform daily activities^35^, and the Short-form Geriatric Depression Scale (SGDS) were used to measure the severity of depression^36^. Before K-CWST, subject were asked whether he or she had discomfort in reading words or colors, and performed a pre-test using 5–6 words using the same setting that the test was performed, to check subjects’ capacity of performing the test. Treatment-emergent adverse events were recorded using the preferred term of the medical dictionary for regulatory activities (MedDRA)^37^. Laboratory measurements included complete blood count and serum panels including electrolytes, creatinine levels, liver enzymes, glucose level, and cholesterol profiles, routine urinalysis results, systolic/diastolic blood pressure and pulse rate, and routine 12-lead electrocardiography results. Serum samples for laboratory evaluations were collected after 8 h of fasting.

### End points

The primary endpoint was set as the change in the total score of the ADAS-cog at 12 weeks from the total score at baseline^5,13,17^. The secondary endpoints were changes in the ADAS-cog subdomain, total ADAS-K, K-CWST, ADCS-ADL, SGDS, and CERAD-K scores.

### Statistical analysis

SAS (Version 9.4, SAS Institute, Cary, North Carolina, USA) was used for the statistical analyses. All analyses to evaluate the drug efficacy were carried out according to the per-protocol principle, and safety analysis was performed using the safety set. Baseline characteristics were compared using Fisher’s exact test for categorical variables or t-tests or Wilcoxon rank sum tests for continuous variables. The change in the scores from baseline to 12 weeks after treatment was evaluated using paired t-tests or Wilcoxon rank sum tests, and inter-group comparisons were performed using analysis of covariance (ANCOVA) and two-sample t-tests. The frequency of adverse events was compared between the groups using chi-square tests or Fisher’s exact test. Post-hoc analysis were performed for the subgroup of 30 subjects with MCI (baseline K-MMSE scores of ≤ 28)^38^. All statistical evaluations were two-tailed, and P values of < 0.05 were set as statistically significant. Power analyses for the minimal sample size to reach a statistical significance were estimated for all of the outcome parameters, using allocation ratio of 1:1, power of 0.8, and α error probability of 0.05. Effect sized was measured using the mean values and the standard deviation of the score changes in the groups.

## Results

**Figure 1:**
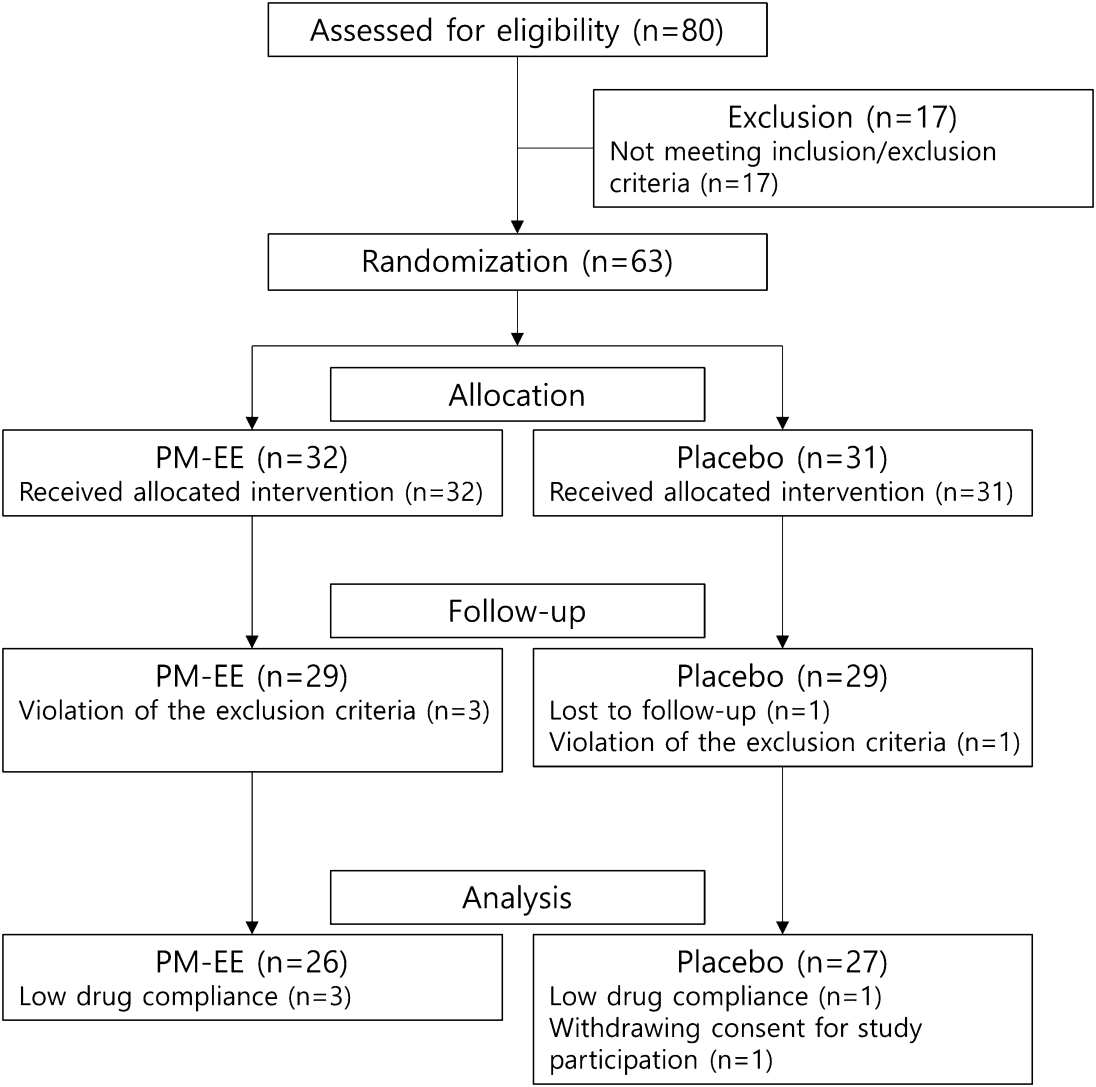
A flow chart illustrating the study process.

Between February 2018 and December 2018, a total of 63 subjects were included in this study. Thirty-two participants were randomized into the PM-EE group, and the remaining 31 were randomized into the placebo group (safety set). During follow-up, 4 subjects (3 in the PM-EE group and 1 in the placebo group) were excluded for violating the exclusion criteria and 1 subject in the placebo group for withdrawing consent for study participation. Four subjects (3 in the PM-EE group and 1 in the placebo group) were excluded from the analysis for low (< 70%) drug compliance and 1 subject for withdrawing consent for study participation (Fig. 1). Finally, 53 participants (26 in the PM-EE group and 27 in the placebo group) were included in the analysis. Demographic and baseline clinical characteristics were comparable between the study groups (Table 1).

After 12 weeks, there were no significant differences in the changes in ADAS-cog scores between the groups (*P* = 0.9858). None of the secondary outcome parameters, such as changes in the ADAS-cog subdomain, total ADAS-K, K-CWST, ADCS-ADL, SGDS, or CERAD-K scores, showed a statistically significant difference between the groups (Table 2). In the power analysis, the total number of study subjects required to reach a statistical significance for the effect of PM-EE on the changes in the ADAS-cog score was 8262. However, when single items of the ADAS-cog were compared, a significant improvement in the comprehension of spoken language items was observed in the PM-EE group (− 0.04 ± 0.20 vs. 0.19 ± 0.56, *P* = 0.0498, Table 3).

**Table 2:** Primary and secondary outcomes at baseline and 12 weeks.

		PM-EE (n = 26)	Placebo (n = 27)	*P* value	Required sample number^¶^ (effect size)
ADAS-K	Baseline	5.74 ± 4.12	6.26 ± 4.36	0.7962^§^	8262
	12 week	4.40 ± 2.68	4.72 ± 3.44		(0.031)
	Change	− 1.35 ± 2.57	− 1.54 ± 3.52	0.9645^†^	
	*P* value^‡^	0.0129*	0.0312*		
ADAS-cog	Baseline	5.71 ± 3.99	6.18 ± 4.31	0.7962^§^	8090
	12 week	4.32 ± 2.49	4.60 ± 3.24		(0.031)
	Change	− 1.39 ± 2.64	− 1.58 ± 3.41	0.9858^†^	
	*P* value^‡^	0.0129*	0.0234*		
ADAS memory	Baseline	4.51 ± 3.19	4.67 ± 3.38	0.8447^§^	4624
	12 week	3.28 ± 2.20	3.23 ± 1.96		(0.041)
	Change	− 1.23 ± 2.34	− 1.43 ± 2.51	0.9008^†^	
	*P* value^‡^	0.0127*	0.0065*		
ADAS language	Baseline	0.96 ± 0.82	1.15 ± 0.95	0.4890^§^	11,172
	12 week	0.96 ± 0.87	1.22 ± 1.58		(0.026)
	Change	0.00 ± 0.75	0.07 ± 1.71	0.8891^†^	
	*P* value^‡^	1.0000	0.8235		
ADAS executive function	Baseline	0.23 ± 0.71	0.37 ± 1.08	0.7167^§^	4974
	12 week	0.08 ± 0.27	0.15 ± 0.36		(0.040)
	Change	− 0.15 ± 0.67	− 0.22 ± 1.05	0.8114^†^	
	*P* value^‡^	0.2560	0.2815		
ADAS-non cog	Baseline	0.04 ± 0.20	0.07 ± 0.27	0.5938^§^	NA
	12 week	0.08 ± 0.27	0.11 ± 0.32		
	Change	0.04 ± 0.20	0.04 ± 0.34	1.0000^†^	
	*P* value^‡^	0.3269	0.5735		
K-CWST word reading valid answer (no)	Baseline	111.04 ± 2.39	111.59 ± 1.55	0.0977^§^	174
	12 week	111.46 ± 2.35	111.59 ± 1.55		(0.209)
	Change	0.42 ± 1.30	0.00 ± 0.48	0.1603^†^	
	*P* value^‡^	0.1099	1.0000		
K-CWST word reading evaluation time (sec)	Baseline	71.65 ± 20.07	79.44 ± 48.80	0.8101^§^	1438
	12 week	64.81 ± 15.01	67.52 ± 20.17		(0.074)
	Change	− 6.85 ± 14.24	− 11.93 ± 46.42	0.7621^†^	
	*P* value^‡^	0.0216*	0.1935		
K-CWST color reading valid answer (no)	Baseline	102.42 ± 21.83	107.26 ± 4.92	0.9214^§^	190
	12 week	108.23 ± 6.72	106.44 ± 11.64		(0.200)
	Change	5.81 ± 20.39	− 0.81 ± 10.31	0.2313^†^	
	*P* value^‡^	0.1588	0.6846		
K-CWST color reading evaluation time (s)	Baseline	139.15 ± 42.91	141.85 ± 45.43	0.9787^§^	408,430
	12 week	125.08 ± 29.51	128.00 ± 34.76		(0.004)
	Change	− 14.08 ± 27.19	− 13.85 ± 25.24	0.6307^†^	
	*P* value^‡^	0.0141*	0.0084*		
ASCS-ADL	Baseline	75.00 ± 0.00	74.96 ± 0.19	0.3454^§^	808
	12 week	74.58 ± 0.81	74.70 ± 0.78		(0.098)
	Change	− 0.42 ± 0.81	− 0.26 ± 0.81	0.3519^†^	
	*P* value^‡^	0.0132*	0.1095		
SGDS	Baseline	3.19 ± 3.29	2.81 ± 3.04	0.7454^§^	180
	12 week	2.12 ± 2.76	2.63 ± 3.24		(0.205)
	Change	− 1.08 ± 2.23	− 0.19 ± 2.00	0.4876^†^	
	*P* value^‡^	0.0208*	0.6346		
CERAD-K word list memory	Baseline	19.46 ± 3.88	19.33 ± 4.50	0.9121^§^	7712
	12 week	23.81 ± 3.51	23.44 ± 4.47		(0.032)
	Change	4.35 ± 4.35	4.11 ± 3.06	0.8204^†^	
	*P* value^‡^	< 0.0001*	< 0.0001*		
CERAD-K word delayed recall	Baseline	8.08 ± 1.49	7.59 ± 1.58	0.3102^§^	11,492
	12 week	8.58 ± 1.58	8.00 ± 2.06		(0.026)
	Change	0.50 ± 1.73	0.41 ± 2.08	0.9927^†^	
	*P* value^‡^	0.1522	0.3182		
CERAD-K word recognition	Baseline	0.00 ± 0.00	0.00 ± 0.00	–	NA
	12 week	0.00 ± 0.00	0.00 ± 0.00		
	Change	0.00 ± 0.00	0.00 ± 0.00	–	
	*P* value^‡^				

The data are reported as the mean ± standard deviation. ADAS-K: Korean version of the Alzheimer's Disease Assessment Scale, ADAS-cog/non-cog: cognitive/non-cognitive domains of the ADAS-K, K-CWST: Korean version of the Color-Word Stroop Test, ADCS-ADL: Alzheimer's Disease Cooperative Study-Activities of Daily Living, SGDS: Short-form Geriatric Depression Scale, and CERAD-K: Korean version of the Consortium to Establish a Registry for Alzheimer's Disease. *P < 0.05, ^§^*P* value for the PM-EE group and the placebo group, by paired t-test or Wilcoxon rank sum test ^†^ *P* value for the PM-EE group and the placebo group, by ANCOVA adjusted by baseline, ^‡^* P* value for the change from baseline, by paired t-test or Wilcoxon rank sum test, and ^¶^ the total number of study subjects required to reach a statistical significance.

**Table 3:** Changes in each item of the ADAS-cog.

		PM-EE (n = 26)	Placebo (n = 27)	*P* value
**ADAS memory**				
Word recall	Baseline	2.55 ± 1.20	2.80 ± 1.59	0.5183^§^
	12 week	2.05 ± 1.42	2.27 ± 1.28	
	Change	− 0.50 ± 1.07	− 0.53 ± 1.42	0.6172^†^
	*P* value^‡^	0.0252*	0.0625	
Orientation	Baseline	0.19 ± 0.80	0.07 ± 0.27	0.9535^§^
	12 week	0.08 ± 0.39	0.04 ± 0.19	
	Change	− 0.12 ± 0.43	− 0.04 ± 0.19	0.5340^†^
	*P* value^‡^	0.1848	0.3265	
Word recognition	Baseline	1.39 ± 1.29	1.42 ± 1.83	0.6862^§^
	12 week	1.15 ± 1.02	0.74 ± 0.69	
	Change	− 0.23 ± 1.20	− 0.68 ± 1.48	0.2839^†^
	*P* value^‡^	0.3365	0.0250*	
Remembering test instruments	Baseline	0.38 ± 0.75	0.37 ± 0.84	0.6096^§^
	12 week	0.00 ± 0.00	0.19 ± 0.62	
	Change	− 0.38 ± 0.75	− 0.19 ± 0.88	0.1384^†^
	*P* value^‡^	0.0152*	0.2835	
**ADAS language**				
Clarity of speech	Baseline	0.00 ± 0.00	0.00 ± 0.00	–
	12 week	0.00 ± 0.00	0.00 ± 0.00	
	Change	0.00 ± 0.00	0.00 ± 0.00	–
	*P* value^‡^	–	–	
Comprehension of spoken language	Baseline	0.08 ± 0.27	0.00 ± 0.00	0.1532^§^
	12 week	0.04 ± 0.20	0.19 ± 0.56	
	Change	− 0.04 ± 0.20	0.19 ± 0.56	0.0498*^†^
	*P* value^‡^	0.3269	0.0961	
Word finding difficulty	Baseline	0.31 ± 0.47	0.41 ± 0.50	0.4600^§^
	12 week	0.38 ± 0.50	0.30 ± 0.47	
	Change	0.08 ± 0.39	− 0.11 ± 0.58	0.1704^†^
	*P* value^‡^	0.3269	0.3265	
Commands	Baseline	0.58 ± 0.50	0.70 ± 0.54	0.4216^§^
	12 week	0.54 ± 0.58	0.67 ± 0.68	
	Change	− 0.04 ± 0.72	− 0.04 ± 0.85	0.9536^†^
	*P* value^‡^	0.7876	0.8235	
Naming Objects and fingers	Baseline	0.00 ± 0.00	0.04 ± 0.19	0.3454^§^
	12 week	0.00 ± 0.00	0.07 ± 0.38	
	Change	0.00 ± 0.00	0.04 ± 0.44	1.0000^†^
	*P* value^‡^	–	0.6632	
**ADAS praxis**				
Constructional praxis	Baseline	0.08 ± 0.27	0.15 ± 0.36	0.4271^§^
	12 week	0.08 ± 0.27	0.11 ± 0.32	
	Change	0.00 ± 0.28	− 0.04 ± 0.44	0.7165^†^
	*P* value^‡^	1.0000	0.6632	
Ideational praxis	Baseline	0.15 ± 0.46	0.22 ± 0.80	0.6995^§^
	12 week	0.00 ± 0.00	0.04 ± 0.19	
	Change	− 0.15 ± 0.46	− 0.19 ± 0.83	0.4668^†^
	*P* value^‡^	0.1034	0.2590	

The data are reported as the mean ± standard deviation. ADAS-K: Korean version of the Alzheimer's Disease Assessment Scale, *P < 0.05, ^§^*P* value for the PM-EE group and the placebo group, by paired t-test or Wilcoxon rank sum test † *P* value for the PM-EE group and the placebo group, by ANCOVA adjusted by baseline, and ‡* P* value for the change from baseline, by paired t-test or Wilcoxon rank sum test.

For the safety analysis, 4 subjects (12.5%, number of events = 7) in the PM-EE group and 4 subjects (12.9%, number of events = 6) in the placebo group reported an adverse event (*P* = 1.000). All of the reported adverse events were of mild severity and were not relevant to the use of the drugs (Table 4). There was no significant difference in the changes in the laboratory parameters from baseline to follow-up between the groups.

**Table 4:** Profiles of adverse events.

		PM-EE (n = 32)	Placebo (n = 31)	*P* value
Severity	Mild	7*	6^§^	1.000
	Moderate	0	0	
	Severe	0	0	
Causality	Definite	0	0	0.070
	Probable	0	0	
	Possible	0	0	
	Unlikely	0	3	
	Unrelated	7	3	

*1 tinnitus event, 2 dyspepsia events, 1 gastroesophageal reflux disease event, 1 irritable bowel syndrome event, 1 herpes zoster skin infection, and 1 trigeminal neuralgia event. ^§^1 dyspepsia event, 1 skin polyp event, 1 herpes zoster skin infection, 1 cervical cyst in the uterus, 1 alopecia areata event, and 1 pruritus event.

In the subgroup analysis for the 30 subjects with baseline K-MMSE scores of ≤ 28 (17 in the PM-EE group and 13 in the placebo group), the baseline profiles of cognitive evaluation scores were comparable between the groups (Table 5). However, the PM-EE group showed higher interval improvement in the color-reading score of the K-CWST (8.2 ± 25.0 vs. − 4.7 ± 13.2, *P* = 0.018). Other parameters, such as ADAS-cog, total ADAS-K, ADCS-ADL, SGDS, or CERAD-K scores, did not exhibit significantly different score changes from baseline between the groups (Table 5). In the power analysis for this subgroup, the total number of study subjects required to reach a statistical significance for the effect of PM-EE on the changes in the ADAS-cog score was 566.

**Table 5:** Subgroup analysis for the 30 subjects with baseline K-MMSE scores of ≤ 28.

		PM-EE (n = 17)	Placebo (n = 13)	*P* value	Required sample number^¶^ (effect size)
ADAS-K	Baseline	6.67 ± 4.81	6.31 ± 4.24	0.7692^§^	470
	12 week	5.20 ± 2.69	5.72 ± 4.20		(0.128)
	Change	− 1.47 ± 2.80	− 0.59 ± 3.90	0.2856^†^	
	*P* value^‡^	0.0455*	0.5957		
ADAS-cog	Baseline	6.61 ± 4.64	6.23 ± 4.21	0.7692^§^	566
	12 week	5.08 ± 2.44	5.49 ± 3.90		(0.117)
	Change	− 1.53 ± 2.90	− 0.74 ± 3.74	0.2949^†^	
	*P* value^‡^	0.0448*	0.4874		
ADAS memory	Baseline	5.31 ± 3.63	4.31 ± 2.29	0.6149^§^	346
	12 week	3.96 ± 2.27	3.64 ± 1.83		(0.149)
	Change	− 1.35 ± 2.61	− 0.67 ± 1.82	0.4257^†^	
	*P* value^‡^	0.0481*	0.2119		
ADAS language	Baseline	0.94 ± 0.90	1.23 ± 1.17	0.5373^§^	810
	12 week	1.00 ± 0.94	1.62 ± 2.02		(0.098)
	Change	0.06 ± 0.83	0.38 ± 2.14	0.7946^†^	
	*P* value^‡^	0.7731	0.5296		
ADAS executive function	Baseline	0.35 ± 0.86	0.69 ± 1.49	0.6532^§^	NA
	12 week	0.12 ± 0.33	0.23 ± 0.44		
	Change	− 0.24 ± 0.83	− 0.46 ± 1.45	0.9794^†^	
	*P* value^‡^	0.2603	0.2735		
ADAS-non cog	Baseline	0.06 ± 0.24	0.08 ± 0.28	0.8845^§^	394
	12 week	0.12 ± 0.33	0.23 ± 0.44		(0.140)
	Change	0.06 ± 0.24	0.15 ± 0.38	0.4208^†^	
	*P* value^‡^	0.3322	0.1654		
K-CWST word reading valid answer (no)	Baseline	110.65 ± 2.89	111.31 ± 2.21	0.1620^§^	154
	12 week	111.18 ± 2.90	111.31 ± 2.21		(0.221)
	Change	0.53 ± 1.59	0.00 ± 0.41	0.3385^†^	
	*P* value^‡^	0.1876	1.0000		
K-CWST word reading evaluation time (s)	Baseline	73.94 ± 23.69	72.31 ± 20.91	1.0000^§^	172
	12 week	65.12 ± 17.58	70.46 ± 27.92		(0.210)
	Change	− 8.82 ± 16.29	− 1.85 ± 16.03	0.5025^†^	
	*P* value^‡^	0.0402*	0.6852		
K-CWST color reading valid answer (no)	Baseline	98.59 ± 26.34	107.69 ± 4.82	0.4100^§^	78
	12 week	106.82 ± 8.01	103.00 ± 16.23		(0.306)
	Change	8.24 ± 25.02	− 4.69 ± 13.24	0.0184^†^*	
	*P* value^‡^	0.1936	0.2256		
K-CWST color reading evaluation time (s)	Baseline	149.82 ± 48.12	140.08 ± 46.85	0.5300^§^	446
	12 week	133.29 ± 29.99	130.92 ± 36.24		(0.132)
	Change	− 16.53 ± 30.17	− 9.15 ± 24.98	0.6152^†^	
	*P* value^‡^	0.0382*	0.2111		
ASCS-ADL	Baseline	75.00 ± 0.00	74.92 ± 0.28	0.2818^§^	2672
	12 week	74.59 ± 0.80	74.62 ± 0.96		(0.054)
	Change	− 0.41 ± 0.80	− 0.31 ± 1.03	0.4796^†^	
	*P* value^‡^	0.0486*	0.3033		
SGDS	Baseline	4.18 ± 3.66	3.62 ± 3.18	0.6634^§^	262
	12 week	2.47 ± 3.30	2.62 ± 3.23		(0.171)
	Change	− 1.71 ± 2.44	− 1.00 ± 1.53	0.7491^†^	
	*P* value^‡^	0.0109*	0.0360		
CERAD-K word list memory	Baseline	18.76 ± 4.04	18.38 ± 4.93	0.8180^§^	13,230
	12 week	22.88 ± 3.02	22.69 ± 5.31		(0.024)
	Change	4.12 ± 3.90	4.31 ± 3.90	0.8958^†^	
	*P* value^‡^	0.0005*	0.0018*		
CERAD-K word delayed recall	Baseline	7.65 ± 1.54	7.46 ± 1.51	0.7437^§^	3998
	12 week	8.35 ± 1.73	8.31 ± 1.97		(0.044)
	Change	0.71 ± 1.69	0.85 ± 1.46	0.8117^†^	
	*P* value^‡^	0.1037	0.0591		
CERAD-K word recognition	Baseline	0.00 ± 0.00	0.00 ± 0.00	–	NA
	12 week	0.00 ± 0.00	0.00 ± 0.00		
	Change	0.00 ± 0.00	0.00 ± 0.00	–	
	*P* value^‡^	–	–		

The data are reported as the mean ± standard deviation. ADAS-K: Korean version of the Alzheimer's Disease Assessment Scale, ADAS-cog/non-cog: cognitive/non-cognitive domains of the ADAS-K, K-CWST: Korean version of the Color-Word Stroop Test, ADCS-ADL: Alzheimer's Disease Cooperative Study-Activities of Daily Living, SGDS: Short-form Geriatric Depression Scale, and CERAD-K: Korean version of the Consortium to Establish a Registry for Alzheimer's Disease. *P < 0.05, ^§^*P* value for the PM-EE group and the placebo group, by paired t-test or Wilcoxon rank sum test † *P* value for the PM-EE group and the placebo group, by ANCOVA adjusted by baseline, and ‡* P* value for the change from baseline, by paired t-test or Wilcoxon rank sum test, and ^¶^ the total number of study subjects required to reach a statistical significance.

## Discussion

This is the first randomized controlled study to investigate the effect of PM-EE in subjects complaining memory dysfunction without overt dementia. The results found that PM-EE did not significantly improve the total score or subdomain scores of the ADAS-cog, K-CWST, ADCS-ADL, SGDS, or CERAD-K. However, in the subpopulation with baseline K-MMSE scores of ≤ 28, PM-EE was associated with significantly more improvement in the color-reading score of the K-CWST. PM-EE also showed significantly improved the comprehension of spoken language items in the language domain of the ADAS-cog. Additionally, PM-EE was safe, without provoking a higher frequency of adverse events, and all of the reported adverse events were of mild severity and were not relevant to the use of PM-EE.

The primary outcome analysis returned a negative result. This finding is in accordance with those from the previous studies that evaluated the disease modifying effects of various candidates in MCI^12–17^. Donepezil, the most established medication for treating AD and other forms of dementia, was not significantly effective in treating MCI and was also associated with a higher frequency of adverse events and a higher rate of drug discontinuation^12,13,17^. Accordingly, this study did not observe a certain trend favouring the effects of PM-EE in the change in the ADAS-cog scores or other outcome parameters. The power analyses also returned very high numbers of participants required to observe a statistically significant effect of PM-EE. This might be due to the participants’ relatively mild or minimal degree of the cognitive dysfunction included in this study, which might have been insufficient to properly evaluate the effect of the drug. However, the number of participants required for a statistical significance of PM-EE in the MCI subgroup (baseline K-MMSE scores of ≤ 28) was comparable to those of the previous studies (250-2100) which evaluated the long-term effects of the candidate drugs in patients with MCI^12–14,16,17^. This implies that in further large-sized studies, PM-EE might be established to be effective in patients with objective cognitive deficits. The properties of the ADAS-cog, the primary outcome parameter of those previous studies and the present study, might also have at least partially contributed to the negative result. A previous pooled analysis of three clinical trials using donepezil in mild-to-moderate AD reported that ADAS-cog components have a substantial ceiling effect (7 out of 11 components)^39^. Therefore, ADAS-cog might be too simple to reflect the expected depth and range of cognitive performance^39^, and might not have been an optimal measurement to evaluate the effects of PM-EE in this clinical setting.

However, we observed a beneficial effect of PM-EE over placebo in improving the comprehension of spoken language function. Language comprehension is a complex cognitive task that requires the simultaneous activation of perception, attention, and language processes and, therefore, is highly implicated in overall cognitive function^40^. A recent study demonstrated that a wide range of brain structures, including the left inferior and middle frontal gyri, left inferior parietal cortex, and left middle temporal gyrus, is activated during the comprehension of spoken language, and their level of activation and functional connectivity decreases with ageing^41^. Therefore, improvement in spoken language comprehension function due to PM-EE might provide a beneficial effect on overall cognitive function.

Notably, PM-EE significantly improved stroop test results in patients with K-MMSE scores compatible with the diagnosis of MCI^38^. The sroop test is a sensitive measurement of the frontal executive function which includes attention, flexibility, cognitive inhibition, and working memory^42^. Frontal executive function is generally diminished in both amnestic and non-amnestic MCI patients, and alterations in executive function is associated with the response to AChE inhibitors^43–46^. A previous study reported that AChE inhibitor activity was up-regulated in the frontal areas of patients with MCI, and the authors assumed this upregulation to be a compensatory mechanism^47^. Additionally, executive dysfunction in MCI and AD has been recognized as a key contributor to patients’ impairment in activities of daily living^48^.

Taken together with the modest improvement in the ADAS-cog in the subgroup with MCI, the effects of PM-EE in improving stroop test results in patients with MCI implies that this drug might be potentially effective in improving the core symptoms of MCI. As disease progression from MCI to dementia takes several years or even a decade^8,10,11^, candidate medications to treat MCI should be safe for long-term use without provoking any harmful effect. Considering the broad mechanism of PM-EE that both enhances AChE inhibitors and has anti-inflammatory, antioxidative, and neuroprotective effects with a favourable safety profile^28^, PM-EE might serve as a potential candidate drug for the long-term treatment of MCI.

In addition to the small number of study population, this study has some limitations to be addressed. First, in previous studies evaluating the effects of various medical candidates in patients with MCI, the change in ADAS-cog scores over time was very slow, and the follow-up duration was 3 years to evaluate the long-term effects of the medication^12–17^. As the current study evaluated the effects of PM-EE after using the drug for only for 12 weeks, a longer duration of follow-up is warranted to properly evaluate the disease-modifying effects of PM-EE in MCI. Second, this study only used a single dose of PM-EE. Because PM-EE was safe without provoking adverse events related to drug usage, future studies might include multiple dosage regimens with higher doses of PM-EE. Additionally, the present study also has limitations as a single-centre study. These limitations might be overcome by further randomized controlled studies with a larger number of patients, a longer follow-up evaluation period, and multiple dosage regimens to demonstrate the long-term clinical effects of PM-EE.

## Supplementary information

Supplementary Information
