# Supplementary material for: Effect of desalted Salicornia europaea L. ethanol extract (PM-EE) on the subjects complaining memory dysfunction without dementia: a 12 week, randomized, double-blind, placebo-controlled clinical trial
Source: Sci Rep. 2020 Nov 16;10:19914. doi: 10.1038/s41598-020-76938-x (PMC7670404; doi:10.1038/s41598-020-76938-x)
Supplement: Supplementary file 1 — Supplementary Information [file 41598_2020_76938_MOESM1_ESM.docx]

Supplemental Materials

**Effect of Desalted *Salicornia europaea L.* Ethanol Extract (PM-EE) on the Subjects Complaining Memory Dysfunction without Dementia: A 12week, Randomized, Double-blind, Placebo-Controlled Clinical Trial**

Woo-Jin Lee, MD^1^, Yong-Won Shin^1^, Da-Eun Kim^1^, Mee-Hyang Kweon, PhD^2^, Manho Kim, MD, PhD^1,3^

^1^ Department of Neurology, Seoul National University Hospital, Seoul, South Korea

^2^ Research Center, Phyto Corporation, Seoul 08826, Republic of Korea

^3^ Protein Metabolism Research Center, Seoul National University College of Medicine, Seoul, South Korea

**Running head:** PM-EE in the subjects without dementia

**Correspondence:**

Manho Kim, MD, PhD

Department of Neurology, Seoul National University Hospital,

101 Daehak-ro, Jongno-gu, Seoul 110-744, South Korea

Tel: +82-2-2072-2193; Fax: + 82-2-3672-7553; E-mail: kimmanho@snu.ac.kr

**List of supplemental Materials: 1 Supplemental Table**

Supplemental Table 1: Contents of the raw materials in PM-EE and Placebo tablets

Supplemental Table 1: Contents of the raw materials in PM-EE and Placebo tablets

| **Materials** | **Content in PM-EE tablet (%)** | **Content in Placebo tablet (%)** |
| --- | --- | --- |
| Desalted *Salicornia europaea L.* extract | 15.00 | - |
| Lactose | 35.00 | 35.00 |
| Crystalline cellulose | 44.82 | 59.82 |
| Magnesium stearate | 1.20 | 1.20 |
| Silicon dioxide | 1.50 | 1.50 |
| Hydroxypropyl Methylcellulose | 1.50 | 1.50 |
| Glycerin fatty acid ester | 0.15 | 0.15 |
| Titanium dioxide | 0.49 | 0.49 |
| Gardenia yellow pigment | 0.14 | 0.14 |
| Gardenia blue pigment | 0.20 | 0.20 |
| **Total** | 100.00 | 100.00 |
